# Design of a Low-Cost Air Levitation System for Teaching Control Engineering

**DOI:** 10.3390/s17102321

**Published:** 2017-10-12

**Authors:** Jesus Chacon, Jacobo Saenz, Luis de la Torre, Jose Manuel Diaz, Francisco Esquembre

**Affiliations:** 1 Departamento de Informática y Automática, ETSI Informatica, UNED, Juan del Rosal 16, 28015 Madrid, Spain; jacobo.saenz@bec.uned.es (J.S.); ldelatorre@dia.uned.es (L.d.l.T.); josema@dia.uned.es (J.M.D.); 2 Departamento de Matemáticas, University of Murcia, 30100 Murcia, Spain; fem@um.es

**Keywords:** distance education, remote laboratories, control engineering education, control systems

## Abstract

Air levitation is the process by which an object is lifted without mechanical support in a stable position, by providing an upward force that counteracts the gravitational force exerted on the object. This work presents a low-cost lab implementation of an air levitation system, based on open solutions. The rapid dynamics makes it especially suitable for a control remote lab. Due to the system’s nature, the design can be optimized and, with some precision trade-off, kept affordable both in cost and construction effort. It was designed to be easily adopted to be used as both a remote lab and as a hands-on lab.

## 1. Introduction

Control engineers need to have both a wide experience implementing solutions in real problems, plants and processes and a deep understanding of the mathematics and theory that lie behind these solutions. Therefore, reaching a balance between theoretical proofs and physical intuition is a major challenge in control education. Lab experimentation plays a key role as a way to connect theory and practice [1,2]. Among others, lab experience serve as [3]:

- an introduction to real world modeling and/or control issues, such as uncertainties, saturation, noise, sensor/actuator dynamics, etc.;
- a demonstration or validation of analytic and theoretical concepts;
- a way of providing facility with instrumentation and measurement tools;
- activities for problem solving and team learning;
- a motivational activity;
- a way to develop professional practices, including maintaining engineering notebooks and report writing.

On the downside, traditional hands-on labs entail high costs related with space requirements, equipment, and maintenance staff [4]. For the last twenty years, there has been a line of research that looks for reducing lab costs by taking advantage of the Internet, i.e., by replacing hands-on labs with online ones. Depending on the nature of the resource (the plant/process), an online lab can be either virtual or remote [5]:

1. *A remote lab* is a real plant that can be accessed through the Internet. Students remotely operate and control a real plant through an experimentation interface.
2. *A virtual lab* is similar to the previous one, but it replaces the physical system with a mathematical model.

Usually, the approach when setting up a new remote laboratory on control is to start from an already working hands-on experiment, and then enable a way to access it from a remote computer: an attractive graphical user interface, security access control, etc. There are many examples of this approach in literature [6,7,8,9,10,11,12,13]. Commercial control experiment systems, however, are expensive, tend to be large and heavy, and in many cases are not very flexible. On the contrary, the teaching needs are subject to changes. For example, a teacher may want to use the system in a level different than the one it was originally designed for. There are many experimentation systems that suffer from lack of use after some years, despite the economical investment. Certainly, after investing hundreds (or thousands) of euros in equipment, it is not easy to discard it simply because it does not fit exactly with our needs.

One of this paper’s goals is to provide a complete open-source and open-hardware remote lab solution that is low-cost and easy to replicate, so that anyone who intends to build it can use it either as a ready-to-use system or as a starting point to introduce custom modifications. For that purpose, there are two essential elements:

- open-source/open-hardware tools;
- rapid prototyping technologies. We have witnessed the rise of 3D printing or single board computers, technologies that fit like a glove to our purposes.

The designs of the 3D printed parts used for building the lab presented in this paper are free to use and modify, so the costs of cloning most of the system’s structural elements is marginal. Moreover, the designs use components that either can be gathered from old electronics devices or are cheap and easy to find. Building a control experiment system from scratch is a demanding and time-consuming task. Sometimes, the use of low-cost components such as sensors or actuators must be compensated with creativity, or loss of performance. However, looking at the success of many community-driven open-source projects (RepRap 3D printers (http://reprap.org/), PublicLab (https://publiclab.org/), Thingiverse (http://www.thingiverse.com/)), it is not unrealistic to think that collaborating between laboratory designers could really enhance the teaching experience in control engineering as well as in other areas.

Given the complementary uses of virtual and real experimentation [14,15,16], the authors have also developed a virtual version of the system. This work presents a low-cost virtual and remote lab implementation of an air levitation system, based on open solutions. It can be easily adopted to be used as both a remote lab and as a hands-on lab. Air levitation is the process by which an object is lifted without mechanical support in a stable position, by providing an upward force that counteracts the gravitational force exerted on the object. In addition to the study of air levitation physics, we found interesting to create a virtual and remote lab of the air levitation system for three reasons:

- the rapid dynamics makes it specially suitable for experiences with a control lab;
- due to the system nature, the design can be optimized and, with some precision tradeoff, kept affordable both in cost and construction efforts;
- the realistic physics of the system are complex and the system is better modeled through an identification process. However, a simple physics model approximation can also be used and compared with the behavior of both the real system and the model obtained through the identification process.

Section 2 offers the guidelines for building the experimental setup and shows the software applications for the real and virtual operation of the system. The models of the air levitation system (the physics one and the identified one) are presented in Section 3. Section 3 and Section 4 present the virtual and remote lab applications and some experiences that can be performed with them, respectively. Finally, Section 5 contains the conclusions of this work.

## 2. The System

The air levitation system presented in this work has a minimalist design because any increase in complexity has an effect in terms of cost and effort and the system is meant to be affordable and easy to replicate. The system is robust enough to work as a remote lab. It is composed of a cylinder in which a forced air flow is used to lift a small object levitating on a desired position.

Before describing the design and construction of the plant, it is worth mentioning that the low cost and optimized design of the system enables two different approaches. Usually, the laboratory creator/maintainer build the system, remote lab, and any other resource needed. Another approach is to let students build their own systems, so they can get a learn-by-doing understanding of a thorough engineering process. Converting an idea or a concept to a practical solution is essential in engineering.

The construction of the system has been decomposed in several tasks:

1. *Design of the experience*;
2. *Design and construction of the plant*;
3. *Design and construction of the server software*;
4. *Creation of the graphical user interface (GUI)*.

The structure is simple on purpose, and there are only a few elements: a methacrylate tube with a nozzle, at one end, coupled with a blower fan. Both elements are supported by an open and movable stand, which let the air flow into the fan. The system has been built using only the following components:

- a methacrylate tube;
- a small and light object;
- 3D printed parts;
- a single-board computer (Beaglebone Black);
- an infrared distance measuring sensor (PIR);
- a PC fan;
- some discrete electronic components and a printed circuit board (PCB);
- a webcam.

All of the design files, documentation about the system and instructions about how to build and setup a new replica is freely available on https://github.com/jcsombria/OpenHardwareLabs.

### 2.1. Hardware

Since the release of the first Raspberry Pi model, a bunch of single board computers have appeared intending to fit developers needs, which range from small do-it-yourself (DIY) projects such as home media centers or domestic appliances, to high performance research computing. Most of these boards are specifically focused on the maker community, students and educators, so they are fully open-source hardware programs.

An interesting feature of these single-board computers is their ability to run a complete Operating System (OS). As an example, a *Raspberry Pi* can run several *Linux* distros (*Raspbian*, *Ubuntu*, *LibreElec*, etc.), Windows 10 IOT Core, or RISC OS, immediately opening an universe of possible applications: it is easy to set up a web server, enable remote connections through Secure Shell (SSH) or even graphical sessions, or use many different programming languages to develop our project. Furthermore, the integrated input/output (IO) capabilities through general purpose input/output (GPIO) pins: digital IO, interconnection protocols (SPI, I2C, etc), or AD converters makes easy (and affordable) to build electronics systems, even if not an expert in the subject.

These single board computers tend to provide similar performance. The most popular ones, like Raspberry Pi or Beaglebone Black, are based on an ARM architecture, presenting differences like the RAM size, input/output capabilities or wireless connectivity. For example, Raspberry Pi provides digital IO, SPI, UART, and I2C connectivity, but it does not have integrated analog IO. On the contrary, Beaglebone Black has analog IO but does not provide built-in WiFi or Bluetooth. Table 1 summarizes the capabilities of four representatives platforms, covering a wide range of costs and functionalities.

The air levitation system presented in this work is controlled by a Beaglebone Black board, running a GNU/Linux distribution. The choice of this board for developing the air levitation system was based on several reasons, namely:

- All boards ship with the Debian GNU/Linux image. This image comes with pre-installed software tools and, in particular, it provides the Node.js runtime and the Cloud9 IDE (Integrated Development Environment). In addition, the bonescript library (included in the Node.js installation) provides an Arduino-like *application programming interface* (API) to access the GPIO, so any person with previous experience in Arduino finds a soft learning curve.
- The GPIO provides analog inputs that can be used to acquire the sensor measures, and PWM outputs to control the fan and the servo of the air levitation system.
- The board has on-board embedded Multi-Media Controller (eMMC) memory, eliminating the need of an external SD card memory.
- There is an active development community. The Beaglebone boards have good hardware/software support and it is easy to find documentation, guides, etc.

It is important to highlight that, while the Beaglebone Black is used in our experimental setup, the other alternatives not only are also suitable but they are entirely compatible with the software and hardware described in the next sections. In fact, since the boards have USB ports, they even can be connected to Arduino boards or other peripherals to add compatibility, reuse other designs or extend the capabilities of the board.

The Beaglebone Black board provides built-in A/D converters to read analog signals. Since the range of the voltage signal provided by the sensor (PIR) lies outside the one admitted by the analog inputs of the board (0,1.8V), it must be adapted before being connected. Similarly, the actuators (fans) require voltages and currents that can not be directly handled by the board, so a signal conditioning circuit has to be used.

Most structural elements have been printed in a *Prusa Mendel i3* 3D printer, a very popular and affordable *RepRap* printer, available at the authors’ department. The 3D parts has been modeled with FreeCAD.

### 2.2. Sensors

**Figure 1:**
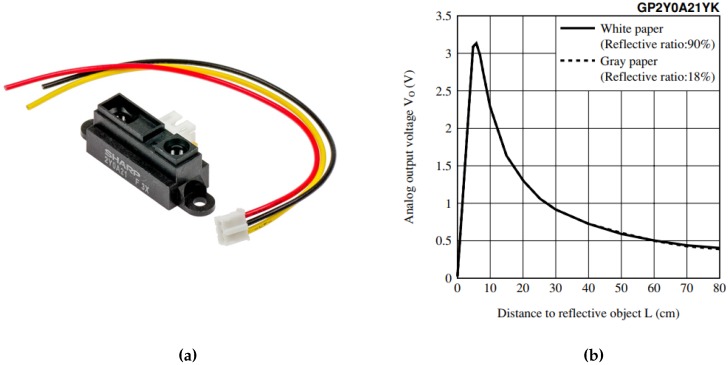
(**a**) Sharp GPY2Y0A21YK infrared distance sensor and (**b**) voltage vs. distance plot.

To measure the position of the ball, several possibilities were considered: visual recognition, ultrasonic sensors, and infrared sensors. While it is interesting to use a video cam to get the ball position, and even could be adequate for teaching in an image processing subject, the complexity and cost of the system would increase, so it was discarded. With respect to the ultrasonic distance sensors, they are a valid alternative as the infrared ones, similar in cost and complexity. However, the latter option was finally chosen. The position of the ball is measured with an infrared beam sensor, particularly a Sharp GP2Y0A21YK0F Analog Distance Sensor (Sharp Corporation, Osaka, Japan), which can obtain measures between 10 and 80 cm. There are other similar models that are electrically compatible and have different ranges, as the GP2Y0A21YK0F (4–30 cm) and the GP2Y0A02YK0F (20–150 cm), so it can be chosen to adapt to different tube lengths. All the aforementioned sensors are analog, yielding a signal roughly in the range (0–5 V), which is proportional to the inverse of the distance measured. The sensor is composed of two IR LEDs, an emitter which projects a light beam, and a receiver that measures the bounce in the detected object. Since the sensor actually measures the light reflected by the object, it may be affected by the color, shape and movement of the object. In addition, it has an update period of approximately 40 ms. These aspects must be taken into account to get a reliable measure. The BeagleBone Black Board has analog inputs that admit a value in the range (0–1.8 V), so the sensor output has to be adapted to that range, which can be done with an op.amp. based circuit. Figure 1 shows the calibration curve corresponding to the GP2Y0A21YK0F sensor.

Since, as mentioned before, the actual map between voltage and position depends on several factors, the sensor must be calibrated with the working conditions. The calibration process was:

- Fix:
  - measurement range ($h_{min}=20$ cm < *h* < $h_{max}=40$ cm);
  - measurement interval (h=1 cm).
- Repeat, for each height (steps of 1 cm):
  - put the ball fixed at a known level;
  - record the sensor voltage for *t* seconds;
  - calculate the mean voltage value and store $v_{i}\to h_{i}$.

**Figure 2:**
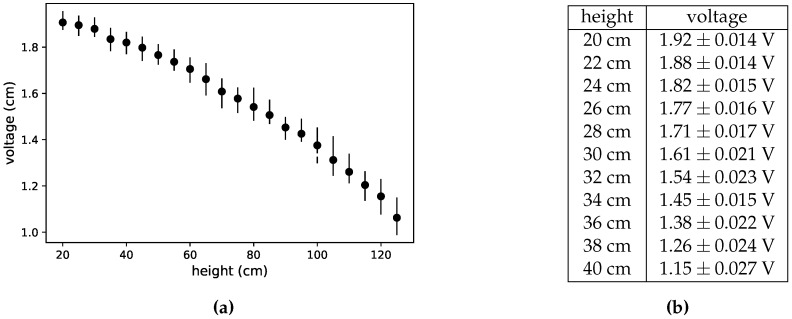
(**a**) Plot of measured voltage vs. distance, and (**b**) measured voltage ranges for some distances.

In a first approximation, there was a problem with a local minimum. Looking at the curve of Figure 1b, it can be seen that for very short distances the voltage grows until around 3 V, and, after that, it monotonically decreases until the maximum distance. This was not the case of the measured response, which decreased at around 15 cm, after that increased until 20 cm, and finally it decreased again. It was a very problematic issue because, in order to avoid that unwanted behaviour, the operating would have to be drastically decreased. After detecting that the strange response was due to the reflection on the tube, the solution adopted was to add two coloured strips inside the tube. Figure 2a shows a plot with the measured voltage vs. distance, and the table in Figure 2b contains the voltage ranges corresponding to some distances.

### 2.3. CAD Software

*Computer Aided Design* (CAD) tools assist the designer to model the physical components that will be part of the system; in our case, the structural parts and the electronics circuits. It is out of the scope of this work to discuss the pros and cons of the so many options available. However, it is worth mentioning at least some of the most popular open-source alternatives that cover the lab needs: FreeCAD, OpenSCAD, KiCad EDA.

FreeCAD is an open-source 3D CAD software tool very popular among the 3D printing community. It has many features, parametric design, multiplatform (works on Linux, Windows and Mac), a fully customizable GUI, and native support for Python scripting and extensions.

OpenSCAD is another popular tool, mostly used to design 3D printed parts. Unlike FreeCAD, it uses a non-graphical with a different modelling approach. It is based on a specific description language, so the creation process is more similar to traditional programming. One of the advantages of this approach is the flexibility to parameterize designs.

The electronic circuits and the PCBs has been created with the software *KiCad EDA* (http://www.kicad-pcb.org), a multiplatform and open-source tool that have the support of the *CERN*, which started the *KiCad EDA* project and have made important contributions to it as part of the *Open Hardware Initiative* (OHI) (https://home.cern/about/updates/2015/02/kicad-software-gets-cern-treatment).

As in the case of 3D printing, there are many PCB manufacturers where you can send your circuit design and have your PCB with professional quality and a moderate cost or, following the maker paradigm, you can build your own circuit with a *computer numerical control* (CNC) PCB milling machine or a chemical etching process.

### 2.4. Design and Construction of the Server Software

The software in the target computer must implement several capabilities, including: *Hardware interface*, *Datalogging*, and *Communication* and *Control* subsystems.

**Figure 3:**
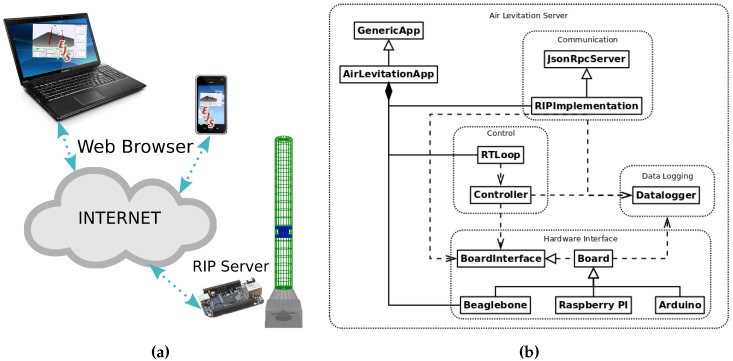
(**a**) Overall system view, and (**b**) RIP software architecture.

The overall picture of the system is represented in Figure 3a,b shows a detailed diagram with the RIP software architecture. The implementations of the aforementioned subsystems map to several Node.js objects, which interact to provide the desired functionality, as follows:

- The *hardware interface* is implemented in *Node.js* by the object *BoardInterface*, which provides a common interface to access the boards, and the boards objects actually implementing the low-level communication. At the moment, only the *Beaglebone Black* and *Arduino* boards have been implemented, but there will be support for other boards in the future. These objects provide several methods to work with the hardware.
- The *datalogging* is implemented by the *Node.js* singleton object *Datalogger*, which gathers the important data and sends it to the database server. Currently, the data can be logged to a local file or sent to an InfluxDB server.
- The *communication* is implemented in Node.js by the object *JsonRpcServer*, which provides basic functionality to create a JSON-RPC 2.0 server, and *RIPServer*, which makes use of the former to implements the API of the *Remote Interoperability Protocol* (RIP). These approach can also be used to easily define new protocols or adapt to other implementations.
- The *control* subsystem is implemented by the object *RealTimeLoop*, which defines the controller implementation.

**Figure 4:**
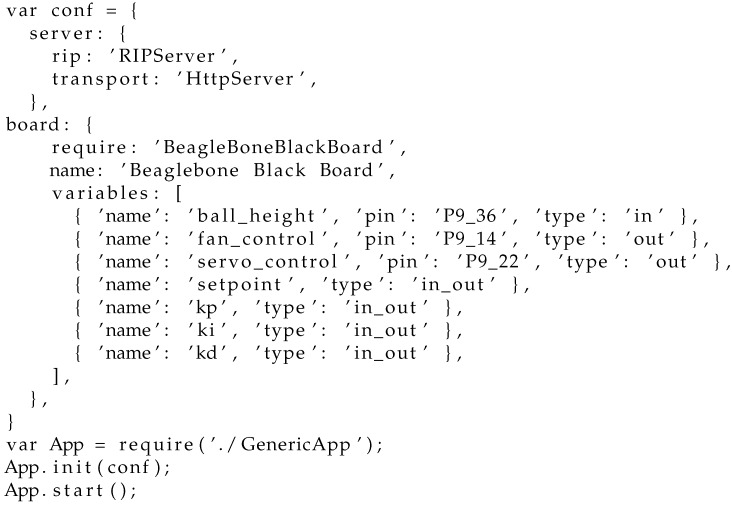
Configuration file.

Since the server software has been designed to be reconfigurable, there will be different implementations of some of the subsystems. In particular, the hardware interface is obviously highly dependent on the hardware, so, in case a different board is used, a new implementation of that part would be needed. To specify which particular implementation should be used, there is a configuration file that allows for creating the experiment by interconnecting components, defining the input and output, transport type, etc. As an example, the configuration of the Air Levitation experience is shown in Figure 4.

The flexibility of the software allows for interoperating with other solutions. One of the reasons to chose Node.js was because it can be easily extended to add new functionality. For example, there are library modules that implement solutions like Profibus, Modbus, MQTT, and many other protocols, and which could be incorporated into our architecture, thus expanding the interoperability.

#### 2.4.1. Hardware Interface

The *hardware interface* purpose is to read measures from the sensors, and send values to the actuators. Though it is obviously very platform dependent, it is a good practice to use standard libraries and protocols. For example, the Arduino API is widely used for its simplicity and it has been exported to other hardware, like the Beaglebone boards or Raspberry Pi. The functionality to be covered can usually be reduced to read and write digital or analog input and outputs.

In the Air Levitation System, the hardware interface task is accomplished using the *bonescript* library, which basically mimics the Arduino API to cope with Beaglebone and the GPIO pins of the board. There is a *real-time loop* implementing the time critical actions: read sensors, update the controller and write outputs. Technically, it is not actually real time because currently it is not supported by the *Node.js bonescript* library. But for the time scale of the system, which is sampled at a 100ms rate, it performs correctly. In case hard real time is needed, there are other alternatives (such as C++) supported by the Beaglebone board.

#### 2.4.2. Datalogging

Once the values have been acquired, it is needed to store them in order to be accessed whenever be required. For that purpose, there are many options, but, again, it is recommended to use a standard solution. There are time series database systems (TSDB) that are specialized on time series management, such as *InfluxDB*, *graphite*, *OpenTSDB* or *RRDtool*. The *datalogging* capabilities have been separated into a low priority task that periodically dumps measures and control actions to a database, so the data is stored and can be accessed to perform offline processing of past sessions.

#### 2.4.3. Communication

The server software, running at the target platform (the single-board computer) must provide an API to interact with the system. The *Remote Interoperability Protocol* (RIP) has been proposed to interconnect engineering systems with user interfaces. It is a simple API based on the JSON-RPC 2.0 protocol which is human-readable and can be easily integrated with *JavaScript* applications, as it uses the *JavaScript Object Notation* (JSON) to encapsulate *remote procedure calls* (RPC). The *communication* subsystem to make the server functionality accessible from outside of the lab computer implements the RIP [17], which provides a standard API to control and monitor the hardware. That basically means that any RIP enabled application can easily interconnect with the server to read and modify variables and plant parameters, so it is easy to decouple the GUI design from the rest of the system.

#### 2.4.4. Control

The remote labs have a local controller implemented, which can be as simple or as sophisticated as needed. In the case of a control engineering lab, it must be a central part of the design, but, even in other cases, it is always necessary to take some safety measures, in order to assure that the system cannot be harmed by accident or by a malicious user. The *control* subsystem implements a *proportional-integral-derivative PID* controller whose parameters can be modified and tuned. The control subsystem is prepare to be extended with more sophisticated controllers without much development effort.

### 2.5. Discussion

Even when the knowledge, money, and time invested in the construction of a remote lab are certainly not negligible, it is an issue that is usually not addressed in literature. When faced with the decision to buy an educational system, several questions may arise, such as why would I spend time and effort in building an experimentation system when I can buy a prebuilt system? Well, there are several reasons that can be argued to justify the decision:

- Commercial academic platforms are very expensive and sometimes can be less flexible. However, they are usually complemented with a curricula of activities, technical support, etc. Building your own system can be cost-effective and the final product is prone to be enhanced or adapted to experiences different from originally thought.
- As mentioned before, the construction process itself is interesting from a didactic point of view. It can be proposed as an academic activity, if not from the scratch, dividing into smaller tasks or with some guidelines so students can address the activity.

In Table 2, an estimation of the cost is provided in working time (hours) and money (€). The first group corresponds to the initial design phase, which is the most difficult and laborious. Here, the results have been measured from the air levitation system developing process. The second group would be the effort needed to replicate existing design and adapt it to our specific needs. The time estimation was calculated as the mean time needed by five people who were given the design resources and told to build the system.

## 3. The Lab

### 3.1. EjsS

EjsS is an open source authoring tool designed to easily create interactive simulations with a GUI for users with no programming skills. EjsS allows users to create applications in both Java or Javascript. Many virtual and remote lab (VRL) applications have been developed using EjsS [6,8,18,19,20,21,22,23,24,25,26] and some of them explicitly define it as a tool that facilitates the development of applications by researcher, teachers and students who want to focus on the simulation theory and not on the technical programming aspects [6,18]. The use of interactive simulations and computer based modelling for teaching physics concepts is described in [20], and a complete discussion of remote labs and their benefits for teaching physics and engineering is available in [22]. Ref. [8] presents a virtual and remote laboratory of mobile robots where EjsS is used in combination with LabVIEW (National Instruments, Austin, TX, USA) and MATLAB (MathWorks, Natick, MA, USA), and, in [18], a new approach to create interactive networked control labs is described. A remote control laboratory based on EjsS, Raspberry Pi and Node.js is described in [21]. The authors of [23] present an ongoing schema to develop virtual models of physical setup equipment and their integration into the corresponding remote laboratory. In [24], students experiment with a set of hands-on exercises about Automatics and Robotics using *RobUALab*, a virtual and remote laboratory developed in EjsS, firstly in face-to-face classes and afterwards accessing the online experimentation environment. Ref. [25] presents the design and implementation of a network for integrating Programmable Logic Controllers (PLC), the Object-Linking and Embedding for Process Control protocol (OPC) and EjsS, and Ref. [26] presents a set of open-source software tools and low-cost hardware architectures are proposed that allow an easy access to local or remote sensors and actuators integrated in EjsS. A systematic approach for developing web-based experimentation environments for control engineering education is presented in [27].

### 3.2. The Virtual/Remote Lab GUI

**Figure 5:**
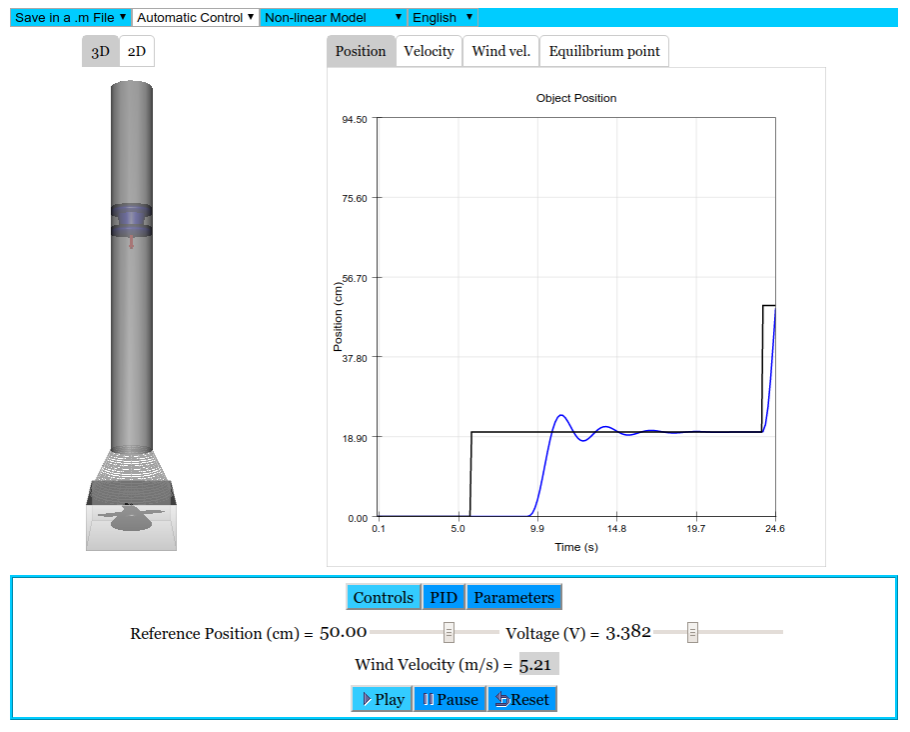
The virtual lab of the air levitation system.

**Figure 6:**
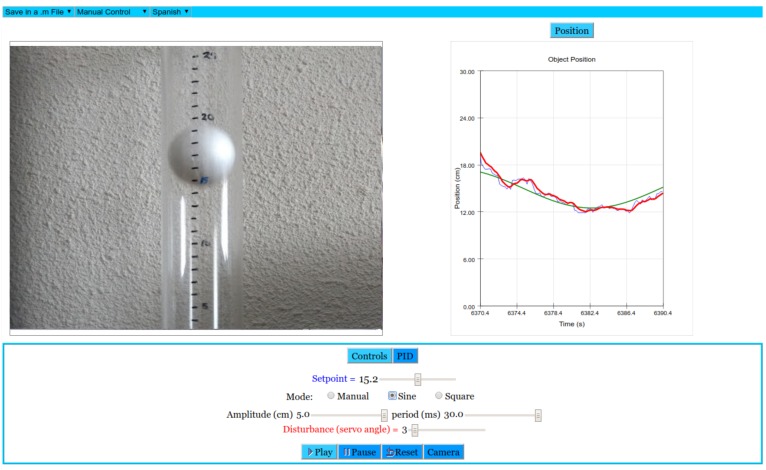
The remote lab of the air levitation system.

As mentioned in Section 1, a nice teaching approach is to provide students with both the virtual and the remote version of an experiment. Both versions of the air levitation system presented here share a simple and clean layout and most of the interface is similar. There is a view of the system on the left (consisting of a video stream obtained from the laboratory webcam in the remote version and on a 3D model in the virtual one). Graphs on the right show the evolution of the interesting variables (the height of the lifting object, the setpoint and the control signal sent to the fan). Finally, there is a control panel at the bottom, which allows for modifying some system parameters, as the controller gains or the setpoint, and the connection buttons, in the remote lab, which are analogous to the simulation execution control ones, in the virtual lab. Figure 5 shows the web interface of the virtual laboratory, and Figure 6 of the remote laboratory.

A description of the options found in the top menu is given below:

- *Save into File*: Used to save graphs and numeric data in a .m file for later analysis.
- *Control Manual/Automatic*: This drop-down list allows modifying the mode in which the experiment runs: open loop or in a closed loop with a controller.
- *Language*: To select between English and Spanish.
- *Linear/Non-linear*: Each of these modes can be selected, corresponding to a linear and nonlinear mathematical model in the virtual laboratory.

## 4. Experiences

Nowadays, there is a great collection of accessible laboratories through internet. Using them, students can learn new concepts and practice with real systems. For this learning to take place, any course with a VRL must also provide a document listing the activities that can be performed with the lab and an user manual.

- The *activities document* contains some guidelines in order to reach the objectives of the lab. Using this guide, the knowledge about systems, control and analysis, the user can obtain fundamental parameters of the system structure or dynamics, information about the best controller configuration, etc.
- The *user manual* facilitates the understanding of the laboratory application’s GUI. It contains information about the available interactions with the interface and a description of the main structure of the views, graphs and menus.

### 4.1. Activities with the Lab

The next paragraphs explain the main activities that can be performed with this lab.

- *PD/PI/PID Tuning*: In Section 4, we said that the user can select an automatic mode, where the system runs into a closed loop. This loop is established for controlling the position of the levitating object inside the tube, which is done by changing the control signal to the fan by means of a PID controller. Using the PID tab in the bottom control panel of the application, the user can modify the proportional, integral and derivative parameters of such controller ($k_{P}$, $T_{I}$, $T_{D}$). The main goal is to be able to tune these parameters so that the output of the system satisfies some required specifications: response time, overshoot, etc. This activity is available in both the remote and the virtual laboratories.
- *Disturbances Analysis*: The virtual and remote labs allow for introducing disturbances in the system. A useful way to obtain this behavior is changing the wind flux outside the fan. In the virtual version, the flux is modified including a random signal in the velocity of the fan. In the real version, this response is obtained using a servo-mechanism similar to a plane flap. The 3D printed flap is designed to divide the base under the fan in two different parts, changing then the local airflow and the flux inside the tube. This possibility of enabling flux disturbances is available in both versions of the lab. In the virtual one, the user can activate the random signal and see how the system response changes. The remote version allows to introduce a single disturbance by changing the angle of the flap using a slider, Figure 6.
- *System Identification*: The system identification is a complex activity in this lab; therefore, the user needs to obtain information from the numeric data gathered in different lab sessions using the virtual and the remote laboratory. In this regard, the students can obtain data files from the GUI with the numeric responses of the system. Then, using a mathematical software program, the students can obtain useful information about the order, poles and zeros of the system.
  - From the simulated version, the user can obtain an initial approach. The virtual laboratory presents two models (discussed in Appendix A): the linear and the nonlinear ones. Using the acquired data in both models, students can obtain information of the system.
  - From the remote lab, and with the knowledge about the virtual air levitation system, the user can try to obtain more information about the real plant in the remote laboratory, where the system identification is not easy.
- *Object Properties Analysis*: The model depends on some parameters like the density of air or the mass and area of the levitating object. In the document of activities, students are encouraged to obtain information about the changes produced in the dynamics when they modify the physical properties of the object in the virtual lab. This activity can be only performed in the simulated version where the physical properties of the object can be easily changed.

## 5. Conclusions

This work presents a low-cost air levitation system to be used in both a virtual and a remote version. This kind of VRL can be used by teachers to complement their classes, giving to their students access to simulated and real resources, which are very important in scientific and engineering areas. These laboratories enhance the knowledge about control experimentation, and the analysis of data to obtain information about systems and the basis in laboratory practices.

## Figures and Tables

**Table 1:** Comparison of low-cost development platforms.

	Onion Omega 2	Raspberry Pi 3	Beaglebone Black	Intel Galileo (Gen 2)
Cost ^1^	20 €	35 €	50 €	75 €
SoC	400 MHz MIPS 24 Kc Big-Endian Processor	Broadcom BCM2837	ARM	Intel Quark SoC ×1000
RAM	64 MB DDR2 (400 MHz)	1 GB LPDDR2 (900 MHz)	512 MB DDR3L (800 MHz)	256 MB DDR3 (800 MHz)
Wireless	WiFi	WiFi, Bluetooth	n/a (WiFi and Bluetooth available with USB dongle)	n/a
GPIO	UART, SPI, I2C, PWM, digital IO	UART, SPI, I2C, PWM, digital IO	UART, SPI, I2C, CAN, PWM, digital IO, A/D inputs	UART, SPI, I2C, JTAG, PWM, digital IO
Ports	USB, WiFi, (more options availaible with expansion boards)	HDMI, 3.5 mm analogue audio-video jack, 4× USB (Universal Serial Bus) 2.0, Ethernet, Camera Serial Interface (CSI), Display Serial Interface (DSI)	HDMI, USB, Ethernet	USB, PCIe, Ethernet

^1^ The prices were checked on www.adafruit.com, www.amazon.com.

**Table 2:** Cost and time estimation.

	Time	Cost
Software	200 h	n/a
Structural parts design	60 h	n/a
Assembling	10 h	<100 €
Lab Software Design	40–100 h	n/a

**Figure A1:**
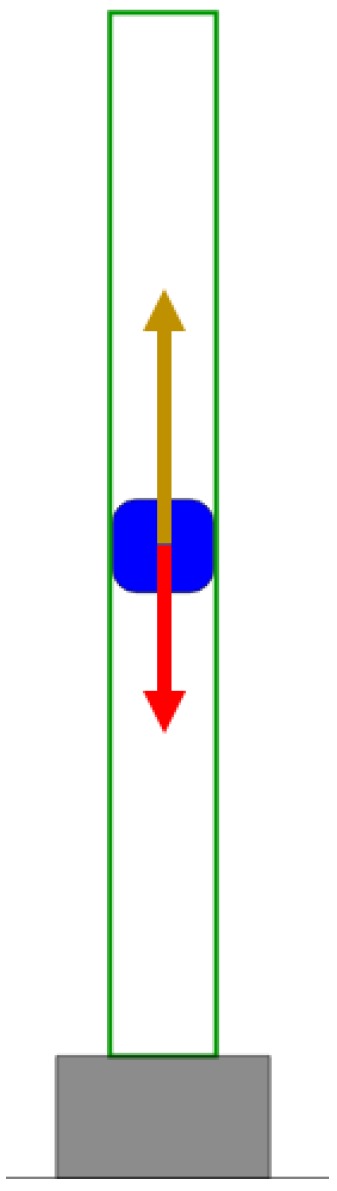
Balance of forces acting over the levitating object.

**Figure A2:**
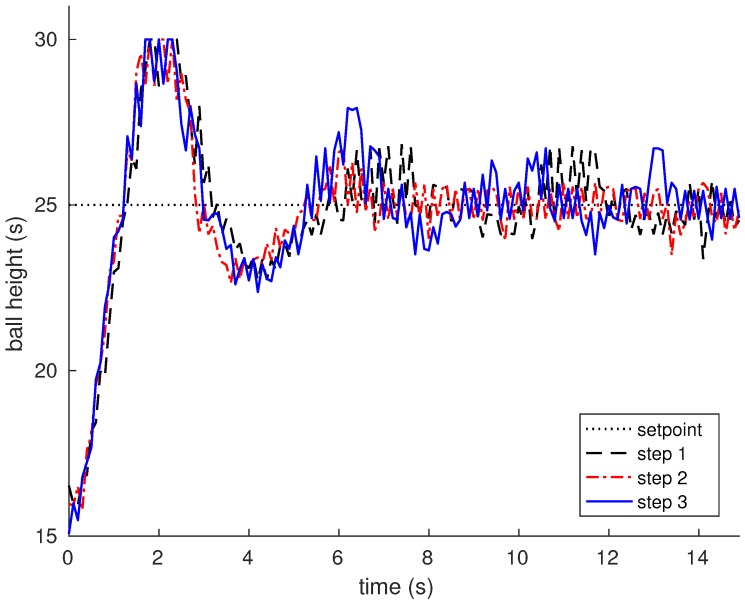
Response to a step input of amplitud 10 cm at the setpoint.

**Figure A3:**
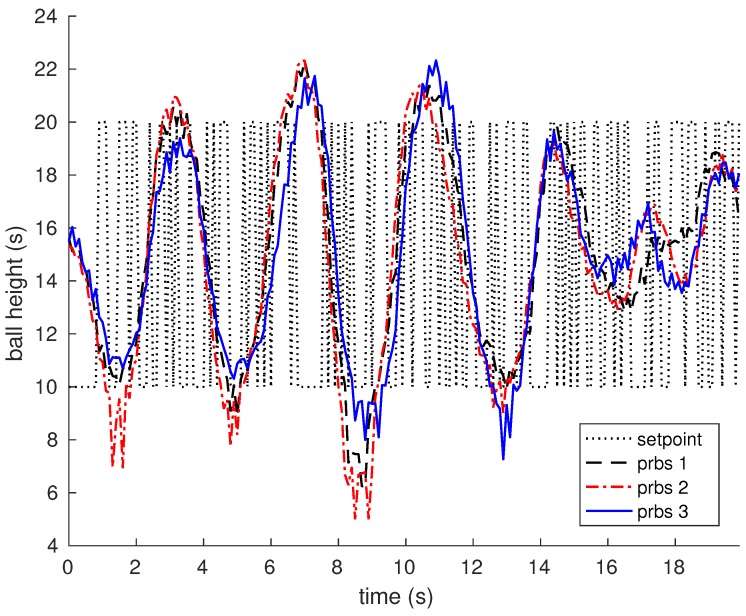
An excerpt of the response to a PRBS (Pseudo Random Binary Signal) input of amplitude 10 cm applied to the setpoint.

**Figure A4:**
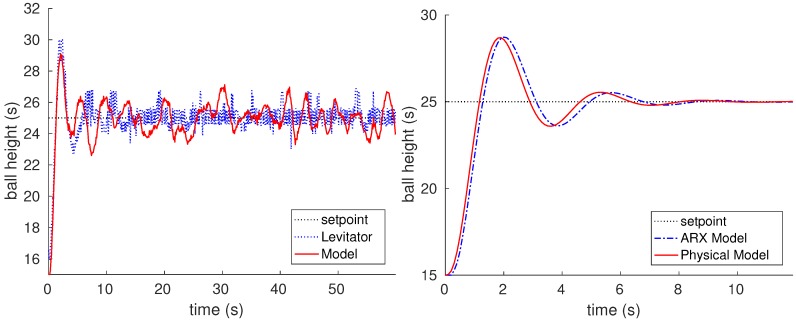
Compared response of the real (dotted line) and identified (solid line) process to a step input of amplitude 10 cm at the setpoint.

## Abbreviations

The following abbreviations are used in this manuscript:

AD	Analog to Digital
API	Application Programming Interface
CAN	Controller Area Network
CAD	Computer Aided Design
CNC	Computer Numerical Control
CSI	Camera Serial Interface
DSI	Display Serial Interface
EjsS	Easy Java/Javascript Simulations
GPIO	General Purpose Input/Output
GUI	Graphical User Interface
I2C	Inter-Integrated Circuit
IoT	Internet of Things
IO	Input/Output
JTAG	Joint Test Action Group
JSON	JavaScript Object Notation
OPC	Object-Linking and Embedding for Process Control
PIR	Passive Infrared Sensor
PLC	Programmable Logic Controller
PWM	Pulse Width Modulation
RIP	Remote Interoperability Protocol
RISC	Reduced Instruction Set Computer
RPC	Remote Procedure Calling
SPI	Serial Peripheral Interface
SSH	Secure Shell
TSDB	Time Series Database
UART	Universal Asynchronous Receiver-Transmitter
USB	Universal Serial Bus
PID	Proportional Integral Derivative
PRBS	Pseudo Random Binary Signal
PCB	Printed Circuit Board
RISC	Reduced Instruction Set Computing
VRL	Virtual and Remote Labs

## Appendix A. Model

Newton’s second law gives us the dynamic equation for the air levitation system, which has been studied by several previous works [28,29,30]. Since the only forces acting over the levitating object are the upwards effect of the air flow (given by the first term in the right part of Equation (A1) and represented by the arrow going upwards in Figure A1), and the downwards effect of the gravity (second term in the right part of Equation (A1) and represented by the arrow going downwards in Figure A1):

(A1) $$m\Delta \ddot{z}=F=\frac{1}{2}\cdot C_{d}\cdot \rho \cdot A\cdot {\left(v_{w}-\dot{z}\right)}^{2}-m\cdot g,$$

where:

- *m* is the mass of the object to levitate;
- *z* is the vertical position of the object in the tube;
- ρ is the density of air;
- *A* is the object’s area exposed to the upwards air flow;
- $v_{w}$ is the velocity of the air inside the tube;
- *g* is the gravity;
- $C_{d}$ is the so-called drag coefficient.

The drag coefficient is a term that depends on Reynold’s number, which, in turn, depends on the relative velocity between the object that moves inside a flow and the velocity of such flow. For small velocities such as the ones considered here, one can assume that $C_{d}$ is constant. In that case, we can express Equation (A1) in terms of a constant named α, being: $\alpha =\frac{1}{2}\cdot C_{d}\cdot \rho \cdot A$:

(A2) $$\ddot{z}=\frac{\alpha }{m}\cdot {\left(v_{w}-\dot{z}\right)}^{2}-g.$$

Now, the levitating object will be in steady state when it does not move, that is, when $\ddot{z}=\dot{z}=0$. Let us call $v_{eq}$ the air speed at such equilibrium point. Then:

(A3) $$g=\frac{\alpha }{m}\cdot v_{eq}^{2}.$$

Therefore, we can finally express the dynamic equation like this:

(A4) $$\ddot{z}=g\cdot \left({\left(\frac{v_{w}-\dot{z}}{v_{eq}}\right)}^{2}-1\right).$$

### Appendix A.1. Linearization

As Section 4 will show, the virtual lab features both the linear and the nonlinear models. Linearization for this system is pretty straightforward. Let $x=\frac{v_{w}-\dot{z}}{v_{eq}}$. Then, Equation (A4) is of the type $f=g\cdot (x^{2}-1)$, and it can be easily linearized around the equilibrium point ($v_{eq}=v_{w}-\dot{z}$, or x=1) using Taylor’s approximation ($f\left(x\right)\approx f\left(1\right)+f^{\prime }\left(1\right)\cdot \left(x-1\right)$):

(A5) $$\ddot{z}=2\cdot g\cdot \left(x-1\right)=\frac{2\cdot g}{v_{eq}}\cdot \left(v_{w}-\dot{z}-v_{eq}\right).$$

### Appendix A.2. Identification

Assuming the system is well described by the linearized model, the process transfer function is:

(A6) $$\frac{\Delta z(s)}{\Delta v(s)}=\frac{1}{s}\frac{a}{\left(s+a\right)},$$

where $a=2g/v_{eq}$, and (Δz, Δv) are, respectively, the increment of the position and wind speed, near the equilibrium point.

Considering the fan can be modeled as a first order process, the transfer function between the input voltage and the wind speed is represented as:

(A7) $$\frac{v(s)}{u(s)}=\frac{k_{v}}{\tau s+1},$$

where and $k_{v}$ is the gain that relates the input voltage to the wind speed at steady state, and τ is the fan time constant that models the impossibility of the fan to change the speed instantaneously.

Finally, the transfer function of the whole process can be stated as:

(A8) $$z\left(s\right)=\frac{1}{s}\frac{ak_{v}}{\left(s+a)(\tau s+1\right)}.$$

Since the model has a pure integrator, the system is unstable in open loop, so a step input is likely to provoke the ball to either fall to the ground or to reach the upper end of the tube. To extract experimental data for identification, after some practical problems with the preliminary experiments, it was decided to identify the closed loop system. The procedure is as follows:

- The operating point is set to $h_{0}=15$ cm;
- The system is identified in closed loop, using a proportional controller with unit gain (u=r−h);
- setting a period of $T_{s}=100$ ms, the system is excited with a signal and 1024 samples are registered. Two kind of signals are applied:
  1. Step (10 cm).
  2. *Pseudo-random bynary signal* (PRBS, ±5 cm).

Figure A2 shows the system response to a step input, repeated three times. The closed loop has a settling time of around 8 seconds, and without steady error due to the pole at the origin. These experiments were carried out as a preliminary approach to obtain an insight on the system parameters such as gain, time constant, etc. in order to design an experience that optimizes the extraction of information about the system.

Figure A3 shows and excerpt of the PRBS signal and the corresponding system response. These experiments provided enough information to obtain an experimental model good enough. The parameters of the model based on physical principles were tuned to fit the experimental data. In spite of that, the model that obtained the best fit to estimation data was an auto-regressive with external input (ARX), with polinomial orders of $n_{a}=4$, $n_{b}=4$ and $n_{k}=3$:

(A9) A(z)y(t)=B(z)u(t)+e(t),

where:

(A10) $$\begin{array}{cc} A(z) & =1-0.714z^{-1}-0.7218z^{-2}+0.07565z^{-3}+0.3468z^{-4}, \end{array}$$

(A11) $$\begin{array}{cc} B(z) & =2.053z^{-3}+1.838z^{-4}+1.379z^{-5}+1.292z^{-6}. \end{array}$$

The model was identified at an operating point $h_{0}=15$, being also valid for $h_{0}=10$ and $h_{0}=20$. The transfer function corresponding to the discrete process can be written as:

(A12) $$G\left(z\right)=\frac{2.053z^{-3}+1.838z^{-2}+1.379z^{-1}+1.292}{z^{-4}-0.714z^{-3}-0.7218z^{-2}+0.07565z^{-1}+0.3468},$$

and the transfer function from the disturbance to the output (for simulation) is modeled as:

(A13) $$H\left(z\right)=\frac{1}{z^{-4}-0.714z^{-3}-0.7218z^{-2}+0.07565z^{-1}+0.3468},$$

where e(t) is white noise with variance σ=0.2798.

The identified model, focused on prediction, obtained an 85% of fit to estimation data. Figure A4 shows the compared response between one of the steps registered with the real system and a simulation of the model, with a disturbance as described by Model A13. It is also interesting to compare the model based on physicals principles with the identified model. Figure A4 shows the response of both models ((A5) and (A12)) (closing the loop with a proportional controller), when an step input is applied to the setpoint.
